# Enteric bacteria boost defences against oxidative stress in *Entamoeba histolytica*

**DOI:** 10.1038/s41598-018-27086-w

**Published:** 2018-06-13

**Authors:** Hugo Varet, Yana Shaulov, Odile Sismeiro, Meirav Trebicz-Geffen, Rachel Legendre, Jean-Yves Coppée, Serge Ankri, Nancy Guillen

**Affiliations:** 1 Institut Pasteur, Plate-forme Transcriptome et Epigenome, Biomics, Centre d’Innovation et Recherche Technologique (Citech), Paris, France; 2 Institut Pasteur, Hub Bioinformatique et Biostatistique, Centre de Bioinformatique, Biostatistique et Biologie Intégrative (C3BI, USR 3756 IP CNRS), Paris, France; 3 Technion Institute, Department of Molecular Microbiology, Ruth and Bruce Rappaport Faculty of Medicine, Haifa, Israel; 4 Centre National de la Recherche Scientifique, CNRS-ERL9195 Paris, France; 5 Institut Pasteur, Paris, France

## Abstract

Oxidative stress is one of the strongest toxic factors in nature: it can harm or even kill cells. Cellular means of subverting the toxicity of oxidative stress are important for the success of infectious diseases. Many types of bacterium inhabit the intestine, where they can encounter pathogens. During oxidative stress, we analyzed the interplay between an intestinal parasite (the pathogenic amoeba Entamoeba histolytica - the agent of amoebiasis) and enteric bacteria (microbiome residents, pathogens and probiotics). We found that live enteric bacteria protected *E. histolytica* against oxidative stress. By high-throughput RNA sequencing, two amoebic regulatory modes were observed with enteric bacteria but not with probiotics. The first controls essential elements of homeostasis, and the second the levels of factors required for amoeba survival. Characteristic genes of both modes have been acquired by the amoebic genome through lateral transfer from the bacterial kingdom (e.g. glycolytic enzymes and leucine-rich proteins). Members of the leucine-rich are homologous to proteins from anti-bacterial innate immune such as Toll-like receptors. The factors identified here suggest that despite its old age in evolutionary terms, the protozoan *E. histolytica* displays key characteristics of higher eukaryotes’ innate immune systems indicating that components of innate immunity existed in the common ancestor of plants and animals.

## Introduction

Human amoebiasis is an infectious disease caused by the amoebic parasite *E. histolytica*. After malaria, amoebiasis is the second most lethal disease caused by a protozoan parasite^1^. Only 10% of infected people develop acute intestinal or extra-intestinal disease^1^. The relationship between commensal or invasive parasite activities may be conditioned (at least in part) by differences in the host’s gut microbiota. However, the mechanisms underlying the molecular switch from a commensal phenotype to a virulent phenotype in *E. histolytica* have not been characterized in detail.

As an obligate parasite in the large intestine, *E. histolytica* occupies a niche with many fellow microbial inhabitants. The parasite feeds on bacteria, and its pathogenicity has been directly linked to exposure to bacterial microbiota^2^. The bacterium-parasite interaction is very selective because only bacteria with the appropriate recognition molecules are ingested by the parasite^3^. Infection with *E. histolytica* has a direct effect on the bacterial populations in the gut microbiota^4^. The presence of specific bacteria (such as enteropathogenic bacteria^5^ and *Prevotella copri*^6^) has been correlated with the presence of *E. histolytica*. In contrast, the presence of segmented filamentous bacteria (for example) is detrimental to the parasite^7^. Furthermore, exposing *E. histolytica* to live *Escherichia coli* O55 boosted the parasite’s cytopathic effect on epithelial cell monolayers *in vitro*^8,9^. Therefore, the gut microbiota may significantly influence the host’s immune response and/or *E. histolytica’*s virulence.

As a parasitic pathogen, invasive amoebiasis is initiated by *E. histolytica*’s penetration of the intestinal mucosa. This amoebiasis is characterized by acute inflammation, with release of pro-inflammatory cytokines, reactive oxygen species and reactive nitrogen species from the host’s activated immune cells. These reactive species are the major cytotoxic effectors for killing *E. histolytica*; they oxidize and nitrosylate amoebic proteins, trigger stress responses, inhibit glycolysis, and reduce the activity of certain virulence factors^10^. Research has shown that *E. histolytica*’s virulence is correlated with its redox power and its expression of antioxidant proteins^11^. *E. histolytica* possess superoxide dismutase (but not-catalases) and redox proteins such as thioredoxins. Three distinct scavenging proteins have been identified in the amoebic proteome: peroxiredoxin, rubrerythrin and hybrid-cluster protein. *E. histolytica* parasite lacks glutathione; its major thiol is L –cysteine, which is known to mediate anti-oxidative defences. Indeed, ROS levels are three times higher in intracellular amoebae cultured in the absence of L-cysteine^12^. The genes encoding ROS scavenger factors do not display significant transcriptional changes upon Oxidative Stress (OS) exposure^13^ - indicating that the mechanism of ROS defence is complex and/or that *E. histolytica* has several protective mechanisms against ROS, which remain to be fully explored. In particular, nothing is known about the influence of intestinal microbiota on the stress response in *E. histolytica*, i.e. whether antioxidant enzymes from bacteria might help the amoeba to defend itself against ROS.

To improve our knowledge on how the anaerobe *E. histolytica* succeeds as a pathogen, it is necessary to understand how the parasite is able to fight against OS. The links between OS, the microbiota and *E. histolytica* have not been characterized. In the present study, we hypothesized that (i) enteric bacteria influence stress responses in *E. histolytica* and (ii) a multifactorial transcriptome analysis might be able to identify the main factors involved in amoebic resistance to OS. Our results showed that OS and live bacteria (LB) together have a profound impact on the *E. histolytica* transcriptome. Nearly 50% of coding genes are affected, and two modes of transcriptional regulation operate together to enable cell survival. Furthermore, we also showed that OS and LB modulate the majority (84 out of 137) of *E. histolytica* genes encoding LRR proteins (including both modes of gene expression) that elicit an antibacterial response. LRR proteins are involved in protein-ligand and protein-protein interactions – especially in proteins that interact with bacterial compounds as part of the innate immune anti-bacterial response in mammals and plants, such as Toll-like receptors (TLRs). Furthermore, we identified striking structural homologies between some human TLRs and amoebic LRRs. Lastly, LRR proteins also include cell surface factors involvement in the interactions between bacteria and human cells.

## Results

### *Escherichia coli* protect *E. histolytica* from OS

**Figure 1:**
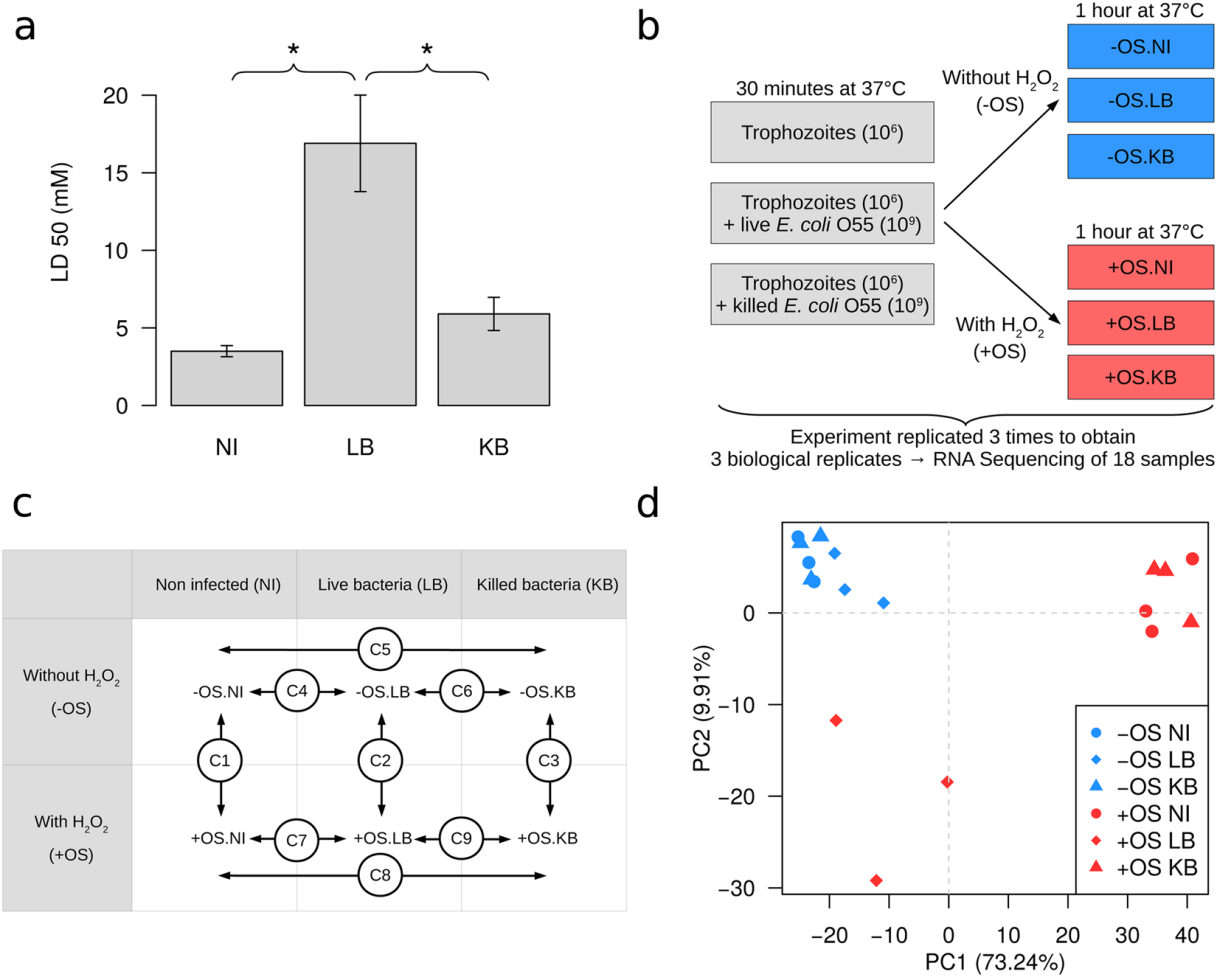
Experimental design and flow chart. (**a**) Determination of the amount of H_2_O_2_ required to kill 50% of the population (LD50). Trophozoites (1 × 10^6^) were treated with (i) H_2_O_2_ for 1 hour (NI); (ii) 1 × 10^9^ live *E. coli* O55 (LB) for 30 minutes and then with H_2_O_2_ for 1 hour; and (iii) heat-killed *E. coli* O55 (KB, the equivalent of 1 × 10^9^ bacteria) for 30 minutes and then with H_2_O_2_ for 1 hour. The trophozoites’ viability was determined in an eosin dye exclusion assay. The values correspond to the amount of H_2_O_2_ (mM) required to kill 50% of the population. Data are displayed as the mean ± standard deviation of three independent experiments that were repeated twice. * indicates P-value ≤ 0.05. (**b**) Experimental procedure for RNA-Seq sample preparation. Trophozoites have been cultured in the absence of *E. coli* (NI), with live *E. coli* (LB) or with killed *E. coli* (KB) and then exposed (or not) to oxidative stress (+OS or −OS). The experiments were replicated three times, leading to the purification and sequencing of 18 RNA samples. (**c**) The biological conditions compared during the differential RNA-Seq analysis. Nine pairwise comparisons are shown: C1 to C3 correspond to the stress effect for NI, LB and KB samples, respectively; C4 to C6 correspond to the effect of bacteria for samples *without* OS, and C7 to C9 correspond to the effect of bacteria for samples *with* OS. The last comparison (C10, not shown) can be considered as the difference between C1 and C2, which was exactly the same as the difference between C4 and C7. (**d**) A principal component analysis performed on the 500 most variable genes in the count data matrix after variance-stabilizing transformation. Biological replicates were grouped together, and the component structure showed that the stress effect was correlated with axis 1 for the NI and KB samples and with axis 2 for the LB samples. The percentage variances associated with each axis are indicated.

To investigate the impact of the relationship between *E. histolytica* - *E. coli* O55 on resistance to OS, we incubated trophozoites *in vitro* with LB or heat-killed bacteria (KB), administered hydrogen peroxide (H_2_O_2_), as a source of OS, and then determined the parasite’s survival rate. We found that trophozoites incubated with live *E. coli* O55 were three times more resistant to OS than trophozoites incubated with heat-killed *E. coli* O55 or not incubated with *E. coli* O55 at all (Fig. 1a). This effect was specific for H_2_O_2_ treatment, since *E. histolytica* incubated with live *E. coli* O55 were just as sensitive to the nitric oxide donor S-nitrosoglutathione (GSNO) as control trophozoites were - suggesting that *E. coli* O55 does not protect *E. histolytica* against nitrosative stress (NS) (Fig. S1).

### Transcriptome responses to the double treatment including *E. coli* and OS

We used RNA sequencing (RNA-Seq) to investigate the molecular bases of *E. coli* O55’s protective effect on *E. histolytica* before exposure to OS (Fig. 1b). RNA-Seq experiments were performed as described previously^14^, and the data were analyzed using the generalized linear model implemented in the DESeq2 package in R^15^. This allowed us to perform pairwise comparisons of gene expression by the *E. histolytica* HM1:IMSS strain under the various test conditions and to probe the interaction between OS and bacteria (Fig. 1c). We first used a principal component analysis to explore the data’s structure (Fig. 1d); this showed that the stress effect is correlated with axis 1 for samples not incubated (NI) with bacteria and samples incubated with KB and with axis 2 for samples incubated with LB. The effect of OS on the *E. histolytica* transcriptome in the presence of LB therefore differs from the effect with KB or NI - suggesting a close interaction between OS and the presence of LB. Our comparisons revealed that in the presence of LB, OS had a profound impact on the *E. histolytica* transcriptome (Table 1). In fact, we observed major changes in transcription levels for several comparisons (e.g. comparison C7), corresponding to nearly 50% of the amoebic genome’s coding regions. Furthermore, the incubation of trophozoites with KB did not affect the transcriptome (see C8, for example).

**Table 1:** Genes modulated in *Entamoeba histolytica* incubated with *Escherichia coli* O55 and experiencing OS.

	Comparison	Modulated genes		
		# down	# up	# total
C1	+OS.NI vs −OS.NI	2412	2314	4726
C2	+OS.LB vs −OS.LB	483	839	1322
C3	+OS.KB vs −OS.KB	2346	2277	4623
C4	−OS.LB vs −OS.NI	35	8	43
C5	−OS.KB vs −OS.NI	0	0	0
C6	−OS.KB vs −OS.LB	1	2	3
C7	+OS.LB vs +OS.NI	1977	2201	4178
C8	+OS.KB vs +OS.NI	1	0	1
C9	+OS.KB vs +OS.LB	2298	2032	4330
C10	(+OS.LB vs −OS.LB) vs (+OS.NI vs −OS.NI)	1757	2021	3778

RNA sequencing and bioinformatics treatments, according to material and methods section, determined the profile of gene transcription. Coding gene models were based on the bona fide gene models defined in previous work^14^.

C: Comparison; OS: Oxidative Stress, LB: Live Bacteria, KB: Killed Bacteria, NI: No Infection.

**Figure 2:**
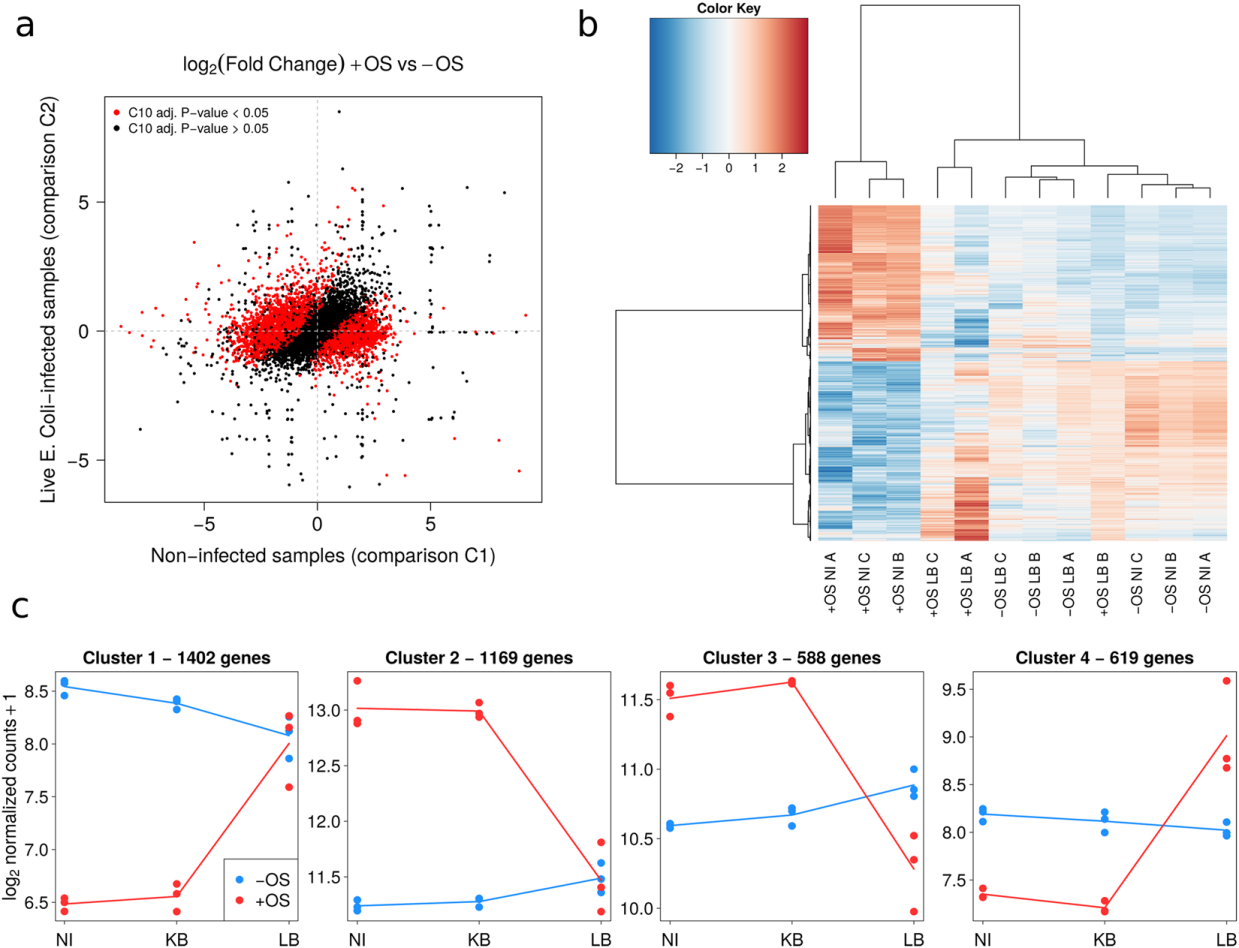
Identification of genes whose expression was modified by incubation with *E. coli* O55 and exposure to OS. On the basis of the transcriptome data, a bioinformatics analysis identified genes under the influence of both OS and LB. (**a**) Illustration of the statistical interaction between OS and LB. A plot of the log_2_(FC) of the stress effect (+OS vs −OS) for LB samples (y-axis, comparison C2) vs. NI samples (x-axis, comparison C1) (Table S1). Red dots correspond to genes with a significant OS-bacterium interaction (comparison C10, n = 3778 with an adjusted p-value ≤ 0.05), i.e. genes with a log_2_(FC) for C1 that differed significantly from the log_2_(FC) for C2. (**b**) A heat map of gene expression values, showing the clustering within the C10 set of genes. Variance-stabilizing transformed counts were used to plot the heat map. The selected genes were associated with a statistically significant stress-bacterium interaction (adjusted p-value ≤ 0.05 for comparison C10, n = 3778). Row/genes values are centred on 0 but not scaled. The genes were hierarchically clustered using the correlation distance and the Ward aggregation criterion. (**c**) Average profiles for the four gene clusters defined by the hierarchical clusters shown in (**b**). The average transcriptome profile was established by computing the mean log_2_-normalized count for the genes in each of the four identified clusters.

We also tested the interaction term of our general linear model, in order to identify genes affected by the combined presence of LB and OS (Fig. 2a). In other words, the effect of OS on these genes differed significantly as a function of the absence of bacteria (comparison C1) or the presence of LB (comparison C2). We identified 3778 genes (Table S1: sheet C10), and then performed a hierarchical clustering analysis (using NI and LB samples) (Fig. 2b). The average profile of each of the four identified clusters was then established (Fig. 2c). Cluster 1 contained 1402 genes, and 98% of these were significantly downregulated in comparison C1. It is noteworthy that 93% of the genes in cluster 1 were not differentially expressed in C2. Cluster 2 comprised 1169 genes; all were upregulated in C1, while 96% were not differentially expressed in C2. Cluster 3 contained 588 genes; as seen for cluster 2, the majority (74%) were upregulated in C1. However, 197 (33%) of the genes in cluster 3 were downregulated in C2 (compared with only 12 (1%) genes in cluster 2 under the same conditions). Lastly, 53% of the 619 genes in cluster 4 were downregulated in C1 and 57% were upregulated in C2. Clusters 1 and 4 thus have similar stress-bacterium interactions, albeit with different proportions of down- and upregulated genes (Table S2).

### Protein classes in each functional category identified upon *E. coli* and OS treatment

The analytical tools in PANTHER (http://pantherdb.org/) were used for functional classification and gene set overrepresentation tests (Table 2).

**Table 2:** Summary of data obtained from diverse bioinformatics treatments of transcriptome clusters analysis.

	OS	OS	Genes	Annotated	Hits	Categories	GO	Unclassified
	NI	LB						
Cluster 1	Down	Normal	1402	498	392	6	28	152
Cluster 2	Up	Normal	1169	735	633	7	36	171
Cluster 3	Up	Down	588	333	272	6	23	76
Cluster 4	Down	Up	619	275	219	5	27	68
All clusters	—	—	3778	1841	1516	—	—	467

**Figure 3:**
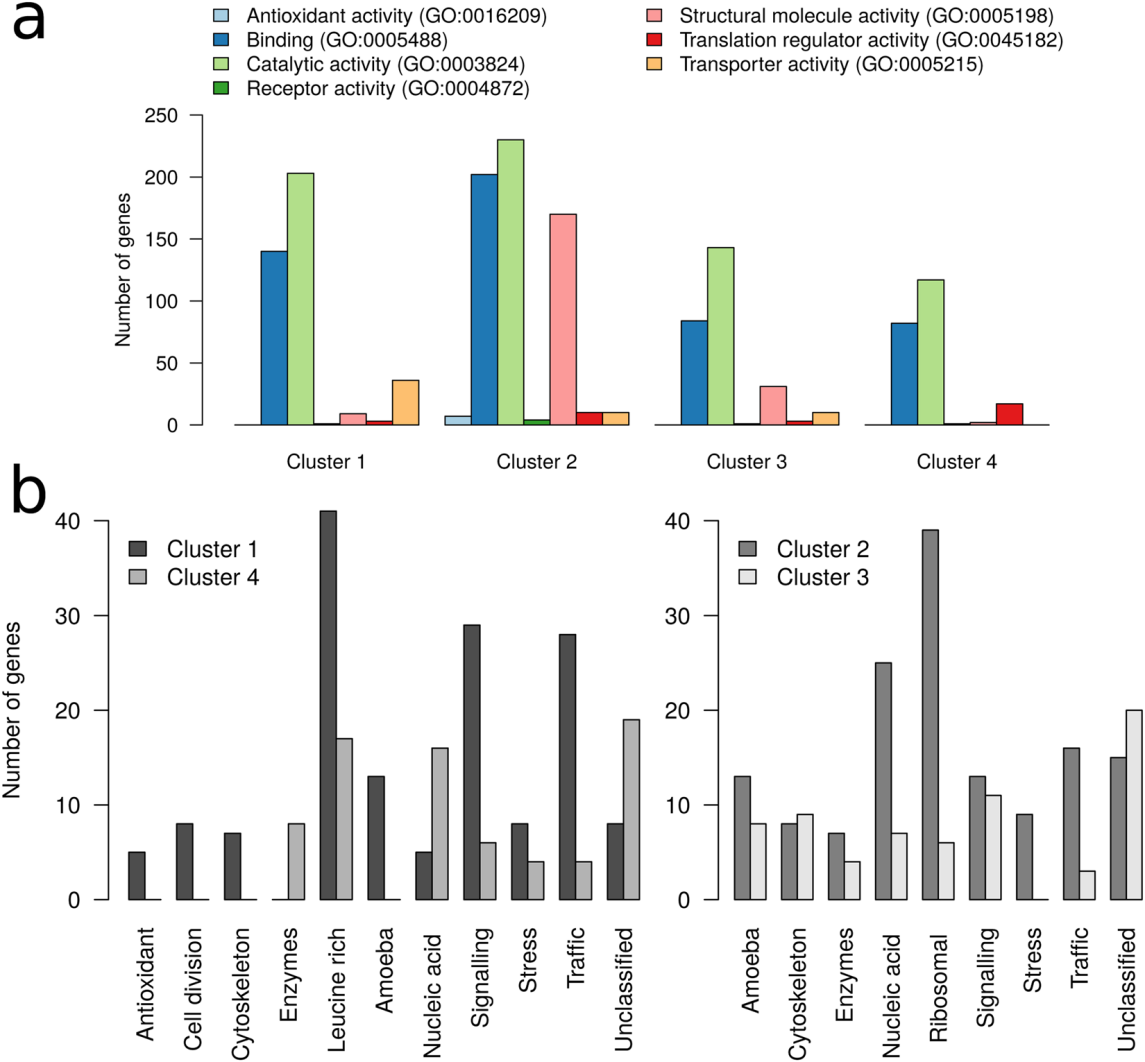
Functional categories and ontologies for the genes in the four identified clusters. (**a**) Functional categories. The number of genes related to each category was derived by processing the data (Table 2) with PANTHER (http://pantherdb.org). The names of the genes in each category are given in Table S3. (**b**) Genes unclassified in the GO term enrichment test. The genes and the corresponding proteins obtained in protein and protein family searches in PANTHER are listed in Table S4.

**Figure 4:**
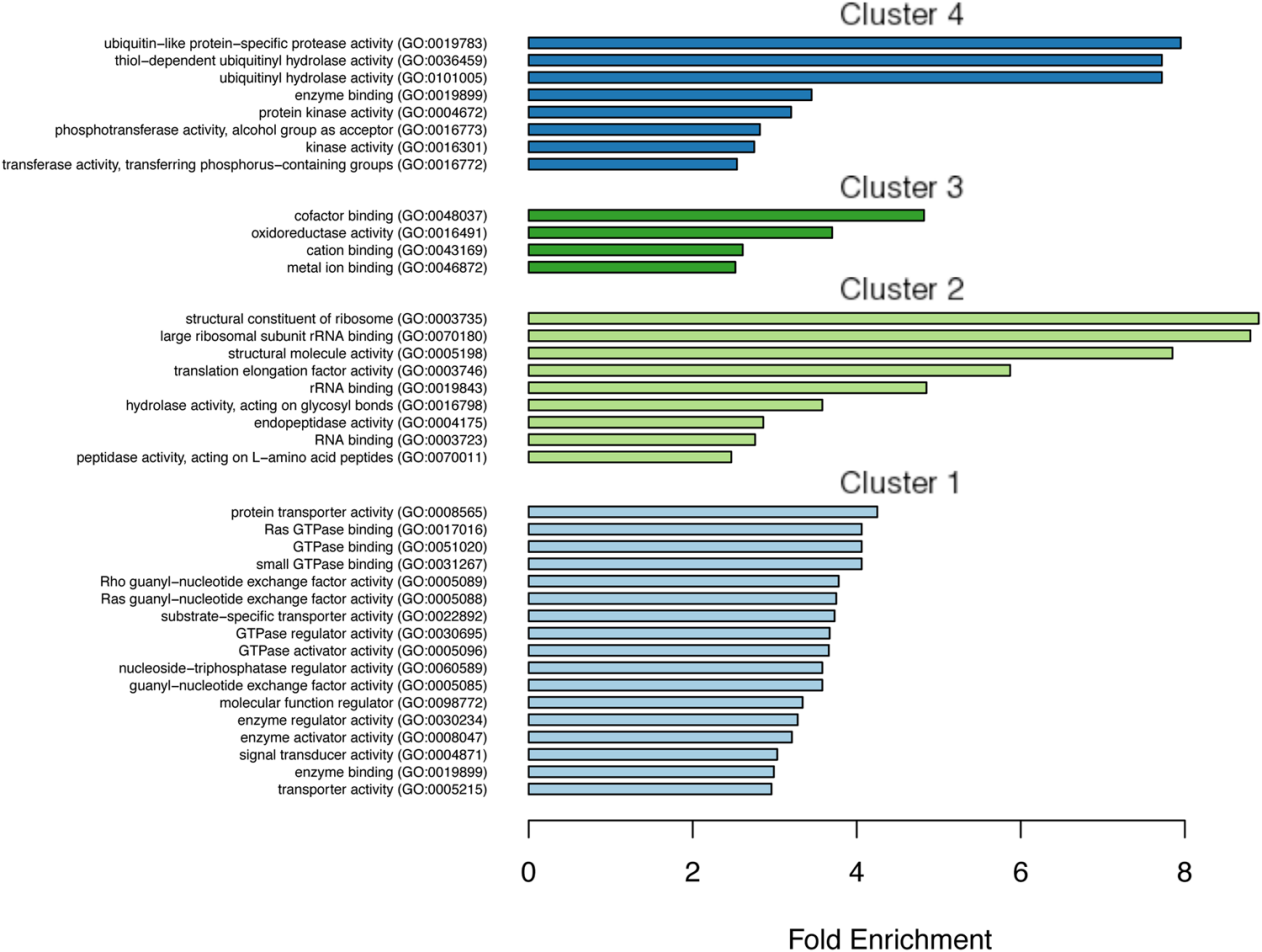
GO term enrichment in the clusters from C10. In cluster 1, the genes that had been downregulated by OS appeared to have normal expression levels in the presence of LB. In cluster 2, the genes that had been upregulated by OS appeared to have normal expression levels in the presence of LB. In cluster 3, the genes were upregulated by OS and downregulated in the presence of LB. In cluster 4, the genes were downregulated by OS and upregulated in the presence of LB. PANTHER tools (http://pantherdb.org/) were used to test the GO term enrichment in gene sets, relative to the background list of all the genes in the amoeba genome. The GO terms were used to identify enriched molecular processes; those with an FE ≥ 2.5 were plotted. A summary for gene ontology (GO) term analysis is provided in Tables 2 and S5.

3778 genes resulting from transcriptome analysis of *E. histolytica* growing (in the presence or absence of *E. coli* O55) and treated by OS (H_2_O_2_ at LD 50) were first identified according to annotations in the amoeba genome at (Amoeba DB: http://amoebadb.org/amoeba/) resulting in 1841 annotated genes that were further submitted to PANTHER classification system (pantherdb.org). The results indicated that 1516 genes match with functional hits from which diverse functional categories were defined (Fig. 3a of main article and Supplemental Table 3). The 1841 genes were then submitted to an overrepresentation test using GO Terms (Fig. 4 and Supplemental Table 4). The genes that do not have a match within the GO terms were also considered (Fig. 3b), as the annotation of amoebic genome is incomplete. OS = oxidative stress by adding H_2_O_2_ at LD dose. NI = non infection; LB = Infected with live bacteria.

To identify the functional categories, we considered only the annotated genes within each cluster (498 in cluster 1, 735 in cluster 2, 333 in cluster 3, and 275 in cluster 4) and used the PANTHER tools for functional classification (which includes entries from *E. histolytica*). This database combines more than 80 genomes. The identified genes fell into seven categories, depending on their predicted activities (Fig. 3a): catalysis, binding, structural molecules, translation, antioxidants, transporters and receptors (Table S3). The categories for functional classification within each cluster (using the subset of molecular functions) are shown in Fig. 3a and Table S3, the data shows the proteins from each category within each cluster. In the binding activity category (for instance), we highlighted many regulators of small GTPases (i.e. guanine exchange factor (GEF) and GTPase-activating (GAP) proteins) in cluster 1, large numbers of genes encoding ribosomal proteins (classes L, S or P) and some small Rho GTPases in cluster 2, several members of the Myb family of DNA binding proteins in cluster 3, and many different GAP and GEF regulatory proteins in cluster 4. In the catalytic activity category, various genes encoding small GTPases, their regulators GAP and GEF, and protein kinases were found in all clusters. In addition, cluster 2 included metabolic enzymes, oxidoreductases and several genes encoding peroxiredoxin (required for the detoxification of H_2_O_2_). We highlighted several metabolic enzymes in cluster 3, including amylases, glycosidase, glyceraldehyde-3-phosphate dehydrogenase, malate dehydrogenase, pyruvate phosphate dikinase, and pyruvate: ferredoxin oxidoreductase. In the structural category, we highlighted the cytoskeletal proteins in cluster 1, with actin-binding proteins (such as cortexillin, formin and villidin) and a gamma-tubulin interactor. In cluster 2, alpha- and gamma-tubulins were identified. The other clusters contained essentially actin and calmodulins. In the antioxidants functional category, we only found peroxiredoxin family proteins (in cluster 2). The results for all other categories were more disperse (Table S3).

### Protein classes highlighted by a Gene Ontology terms analysis of each gene cluster

We restricted our analyses to the families of factors enriched more than 2.5 fold in the overrepresentation test. Not all of *E. histolytica*’s genes have an equivalent or are well annotated in the PANTHER family and protein class database. Consequently, we also considered genes that were not classified in the overrepresentation test (Fig. 3b and Table S4).

#### Functional analyses of proteins in clusters 1 and 4

The bioinformatics analysis of genes present in cluster 1 (Table 2, Fig. 4 and Table S5) showed significant enrichment in 28 GO terms; it highlighted proteins with transporter activity GO:0008565 (Fold Enrichment, FE: 4.3), including factors involved in the endomembrane traffic of *E. histolytica* (e.g. clathrin, Vps35, VpS26, and AP-beta) or nuclear transport (e.g. Ran binding protein and nuclear transport receptors). Many small GTPases from the Rho family GO:0017016 (FE: 4) were also found, together with their regulators RhoGEF or RhoGAP GO:0005089 (FE: 3.6); these appeared in several combinations in the GO term enrichment analysis (Fig. 4). Cluster 1 was enriched in kinases, including the serine/threonine or tyrosine kinase identified in GO:0004871 (FE: 3).

Genes in cluster 4 were significantly enriched in 27 GO terms, of which 8 presented an FE ≥ 2.5 (Table 2, Fig. 4 and Table S5). Ubiquitination of proteins leads to their degradation in the proteasome - particularly under stress conditions. The most overrepresented proteins were ubiquitin proteases GO:0019783 (FE: 8) and ubiquitin hydrolases GO:0036459 (FE: 7.7) and GO:0101005 (FE: 7.7), which remove ubiquitin from proteins. The enzymes in GO0019899 (FE: 3.5) essentially correspond to RabGTPases, and 28 entries were in the protein kinase class GO0004672 (FE: 3.3).

#### Functional analyses of proteins in clusters 2 and 3

The signature of genes found in cluster 2 GO terms (36) correspond to huge number and highly significant FE for structural constituents of ribosomes; GO0003735 (FE: 9), GO:0070180 (FE: 8.8) and GO:0005198 (FE: 7.8) all with 122 entries identified (Table S4). Translation elongation factors GO:0003746 (FE: 5.9), ribosomal RNA binding GO:0019843 (FE: 4.8). Cluster 2 additionally contained proteins with hydrolase activity, acting on glycosyl bonds (including beta amylase) GO0016798 (FE: 3.6) and cysteine proteases GO0004175 (FE: 2.8). In cluster 3, 23 GO terms were enriched: cofactor binding (GO:0048037) (FE: 4.9), oxidoreductase activity GO:0016491 (FE: 3.7) including enzymes from glycolytic pathways, cation binding GO:0043169 (FE: 2.6) and metal ion binding GO:0046872 (FE: 2.5) had the highest FEs (Fig. 4, Table S5).

#### Genes in each cluster not classified by the overrepresentation test

Examinations of the genes not identified by GO terms but present in cluster 1 revealed RhoGAPs, signalling molecules (such as phosphatases), thioredoxin (involved in cell responses to reactive oxygen species), proteins involved in the endomembrane traffic, and factors related to the cytoskeleton, stress, membrane association, the cell cycle and nucleic acid processing (Fig. 3b and Table S4). We found a large number (41) of genes encoding Leucine-rich repeat (LRR) proteins. In cluster 4, several diverse protein classes were found (e.g. those interacting with nucleic acids, associated with trafficking or related to stress), with 17 new entries in the LRR protein family. The identified categories were similar in clusters 2 and 3; they included genes for proteins related to stress, the cytoskeleton, and nucleic acid processing, as well as many genes encoding ribosomal proteins and a few enzymes. Signalling, trafficking and metabolic pathways were also abundant. Some *Entamoeba*-specific proteins were found, including Ariel, proteins involved in cyst formation, the serine-rich antigen, the light subunit of the galactose lectin, and one entry from the LRR protein family.

### Two distinct gene responses occurs in *E. histolytica* in the presence of LB and OS

**Figure 5:**
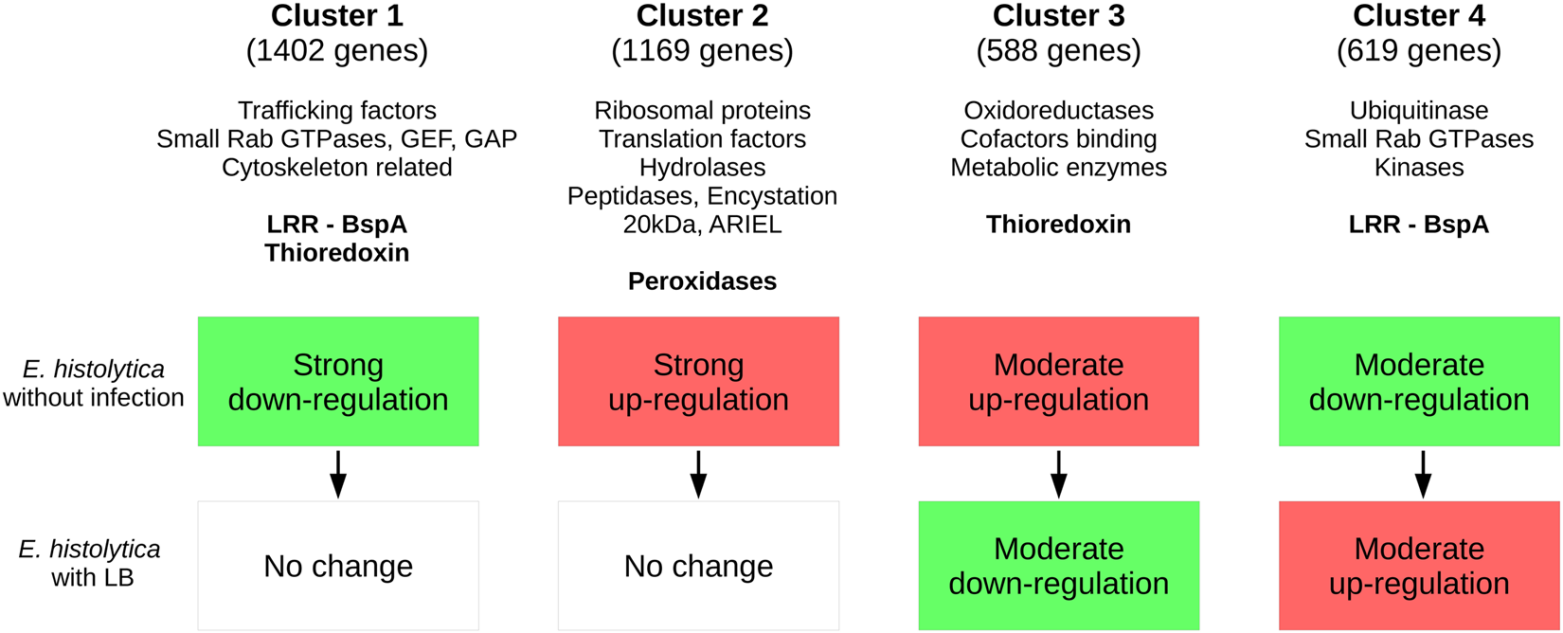
Summary of *E. histolytica*’s gene responses to OS in the presence of *E. coli* O55, as a function of the clusters defined in Fig. 2b.

Our bioinformatics analysis revealed that two patterns of gene regulation were significantly involved in the massive response to OS. The first pattern corresponds to genes for which the intensity of OS-induced changes in expression is reduced by pre-incubation with LB (cluster 1 and 2). This pattern accounts for approximately 31% of the coding sequences in the amoeba genome, and includes genes involved in protein synthesis and homeostasis (ribosomal proteins and translation-related factors), nutrition (hydrolases, peptidases and trafficking) and survival (encystation factors), as well as specific amoebic proteins of unknown function (including ARIEL and members of the LRR protein family). Genes for several types of antioxidant enzyme (e.g. peroxidases and thioredoxins) are influenced in this manner ((Fig. 5 and Table 3).

**Table 3:** Antioxidant enzymes.

CL	Mapped ID	Name	Family/Subfamily	Protein class
1	EHI_133970	Thioredoxin	Non HIT	Non HIT
1	EHI_107670	Thioredoxin	Non HIT	Non HIT
1	EHI_152600	Thioredoxin	Non HIT	Non HIT
1	EHI_124400	Thioredoxin	Non HIT	Non HIT
1	EHI_190880	Thioredoxin	Non HIT	Non HIT
2	EHI_001420	Peroxiredoxin	AT16346P-RELATED/(PTHR10681:SF135)	PR(PC00180)
2	EHI_084260	Peroxiredoxin	AT16346P-RELATED/(PTHR10681:SF135)	PR(PC00180)
2	EHI_122310	Peroxiredoxin	AT16346P-RELATED/(PTHR10681:SF135)	PR(PC00180)
2	EHI_121620	Peroxiredoxin	AT16346P-RELATED/(PTHR10681:SF135)	PR(PC00180))
2	EHI_145840	Peroxiredoxin	AT16346P-RELATED/(PTHR10681:SF135)	PR(PC00180)
2	EHI_123390	Peroxiredoxin	AT16346P-RELATED/(PTHR10681:SF135)	PR(PC00180)
2	EHI_106330	Nitroreductase	IODOTYROSINE DEHALOGENASE/(PTHR23026:SF120)	PR(PC00180)
3	EHI_125740	Oxidoreductase	BILIVERDIN REDUCTASE A/(PTHR43377:SF4)	DHY (PC00092)
3	EHI_146380	Oxidoreductase	NOT NAMED/(PTHR24320:SF121)	DHY (PC00092)
3	EHI_170420	Thioredoxin	Non HIT	Non HIT

In cluster 1 the genes that had been downregulated by OS appeared with normal expression levels in the presence of LB. In cluster 2, the genes that had been upregulated by OS appeared with normal expression levels in the presence of LB. In cluster 3, the genes were upregulated by OS and downregulated in the presence of LB. In cluster 4, the genes were downregulated by OS and upregulated in the presence of LB. The set of genes was submitted to PANTHER tools (pantherdb.org) to identify the family and protein class. From there the antioxidant proteins were extracted. CL: Cluster, PR: peroxidase, DHY: dehydrogenase.

The second pattern corresponds to genes for which the direction of OS-induced changes in expression (upregulation or downregulation) is reversed by pre-incubation with LB (Fig. 5) (clusters 3 and 4). This pattern accounts for approximately 14% of the coding sequences in the amoeba genome, and includes genes for deubiquitinating enzymes (which cleave ubiquitin from protein substrates and regulate protein degradation), oxidoreductase enzymes (e.g. alcohol dehydrogenase, malate dehydrogenase, and pyruvate:ferredoxin oxidoreductase) and new 17 alleles from the LRR family. The present study also shows that OS modulates the expression of *E. histolytica’s* LRR family of proteins as a whole.

### Gene responses in *Entamoeba histolytica* incubated with several enteric bacteria and treated by OS

The specificity of *E. histolytica*’s transcriptomic response to *E. coli* and OS was further investigated by incubating trophozoites (prior to H_2_O_2_ treatment) with other bacteria, including S*almonella enterica* (an enteropathogen often encountered in amoebic coinfections), *Enterococcus faecalis* (a member of the human microbiota) and *Lactobacillus acidophilus* (a popular probiotic). *E. coli* was used as the control. We found that *E. coli*, *S. enterica* and *E. faecalis* (but not *L. acidophilus*) protected *E. histolytica* against OS (Fig. S2). In fact, pre-incubation with *L. acidophilus* was associated with a highly specific but non-protective response that involved some major signalling molecules, such as kinases, regulators of small GTPases and oxidoreductases (Table S6) and with high significance were the genes encoding proteins necessary for ribosome structure (Fig. S2). Relative to the experiment with *E. coli*, the transcriptomic changes induced by exposure to OS were similar for live *E. faecalis* and (to a lesser extent) *S. enterica* and the results again highlighted the set of genes encoding LRR factors (84 out of 137) (Table S6).

### LRR proteins as a key element of *E. histolytica* responses to bacteria and OS

**Figure 6:**
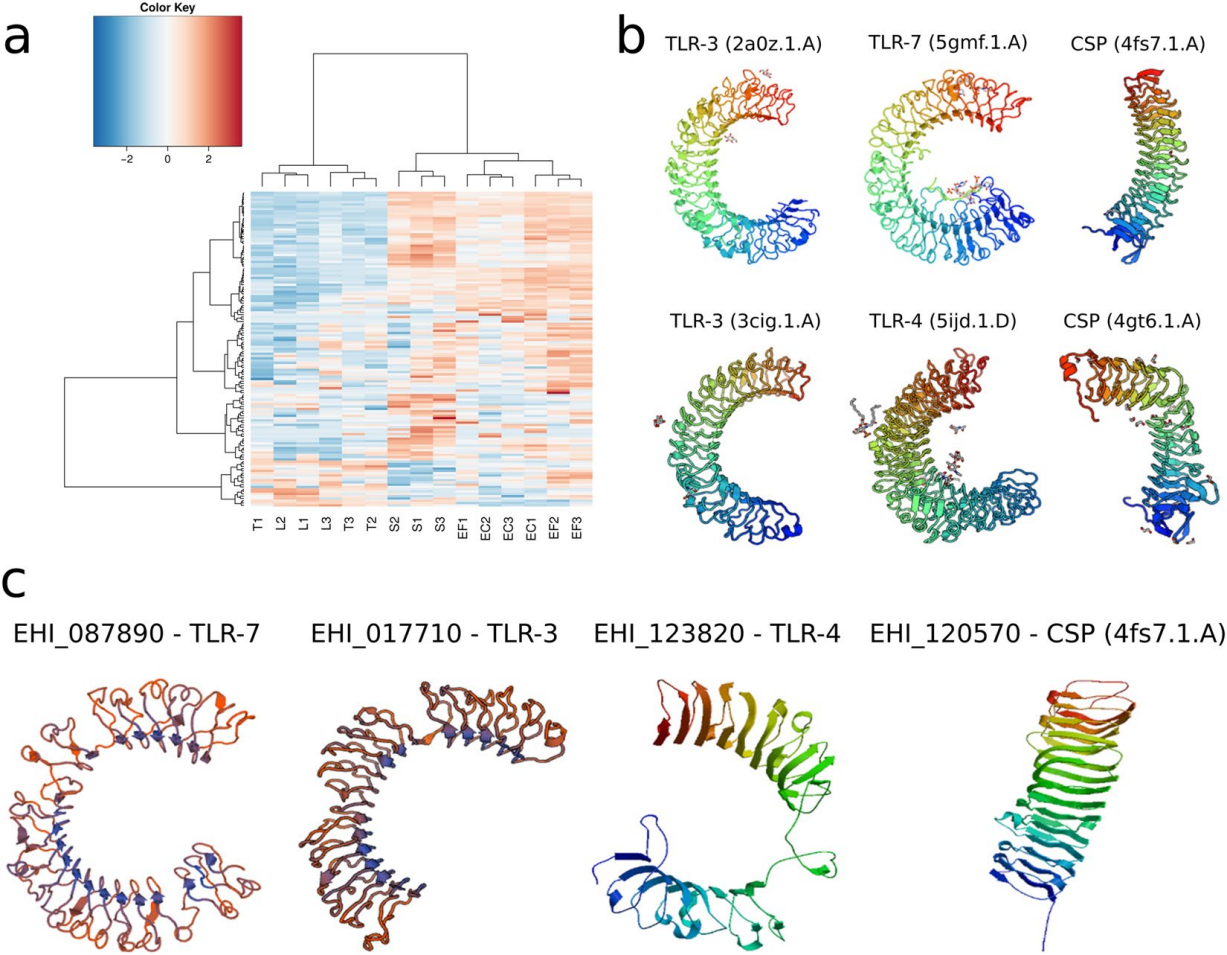
*E. histolytica* transcriptome changes in the presence of E. coli, E. faecalis, S. enterica or L. acidophilus. On the basis of the transcriptome data (Supplemental Table 6), categories and GO term enrichment (Fig. S2) were analyzed with PANTHER (http://pantherdb.org) for all modulated genes. Genes encoding LRR proteins were extracted from this analysis. (**a**) Heat map of gene expression values for the genes encoding LRR proteins. Variance-stabilizing transformed counts were used to draw the heat map, and row/genes values were centred on 0 but not scaled. The genes in the clusters and the corresponding changes in gene expression are listed in Supplemental Table 7. (**b**) Structural protein models. After selection of the genes displaying the greatest transcriptome changes, encoded proteins were fed into a two-dimensional analysis (Supplemental DOC1) and 3D structural analysis (Table S8). The 3D structure for several templates (according to the Protein Data Bank) is shown, including 4fs7.1.A (BspA protein from *Bacteroides ovatus*), 3cig.1.A or 2a0z.1.A (Toll-like receptor 3); 5gmf.1.A (Toll-like receptor 7) and 5ijd.1.D (Toll-like receptor 4, variable lymphocyte receptor B chimera). The SWISS-MODEL template library was searched with Blast and HHBlits for evolutionarily-related structures that matched the target sequence, and the corresponding 3D structural models were identified (Supplemental Table 8). (**c**) 3D amoebic protein models were built using the templates indicated in B. The ID of the *E. histolytica* protein is shown with the modelling template.

The intense changes in the expression of the LRR family genes described above prompted us to perform an in-depth analysis of transcription changes and protein structures. A hierarchical clustering analysis of the expression data enabled us to separate the 137 genes into three groups: group 1 contained 88 genes that were greatly overexpressed in *E. coli*, *E. faecalis* and *S. enterica*, group 2 comprised 20 genes that were underexpressed in these bacteria, and group 3 encompassed 29 genes that were particularly overexpressed in the presence of *S. enterica* (Fig. 6a and Table S7). Given that proteins containing LRR motifs cover a large range of biological functions^16,17^, we choose the 10 most strongly expressed genes from each group. After removing duplicates, we considered 14 LRR proteins corresponding to the most significantly modulated genes. A protein domain search and three-dimensional (3D) protein structure modelling revealed a striking homology with the ectodomain of toll-like receptors (TLRs) for 11 of the 14 candidates^18,19^, with particularly high scores for TLR-4 (EHI_123820 and EHI_119470), TLR-13/-3 (EHI_017710, EHI_139980) and TLR-7/-9 (EHI_087810). TLR-4 is a key factor in the recognition of lipopolysaccharides from Gram-negative bacteria^20^. TLR-3, -7 and -9 recognize nucleic acid sequences^18^, However, the amoebic proteins with homology to TLRs do not bear transmembrane domains or the Toll/interleukin-1 receptor (TIR) domain required for signalling. Six other proteins displayed equally striking homology with bacterial BspA cell surface proteins or with TLR-3 (Fig. 6b,c, Table S8). For three of the proteins, only homologies with bacterial BspA proteins were found. BspA proteins are involved in bacterial adhesion to cells or to the extracellular matrix^21,22^.

## Discussion

### By regulating gene expression, bacteria have a powerful protective effect on *E. histolytica* exposed to OS

Oxidative stress occurs when excess levels of intracelullar ROS overwhelm the cell’s normal antioxidant capacity. The excess ROS are neutralized by antioxidant compounds (e.g. reduced thiols and glutathione) and/or enzymes involved in protective ROS scavenging systems (superoxide dismutase, peroxidase, catalase, and glutathione reductase). When high levels of ROS are not controlled by the intracellular defence mechanisms, the oxidative damage to proteins, lipids, and DNA can lead to cell death. ROS production is a powerful component of the innate immune defence against microbial infections, including amoebiasis^23^. Since *E. histolytica* is an anaerobic parasite, the trophozoite must maintain low intracellular ROS levels during the intestinal invasive process if it is to avoid the host’s immune attack. The reduction of O_2_ and H_2_O_2_ and the production of antioxidants are required amoebic virulence^24^. In the context of infection, *E. histolytica* interacts with both the microbiota and the human host. The present study concerned the changes in the *E. histolytica* transcriptome induced by OS and live Enterobacteriaceae. To the best of our knowledge, our study is the first to show that LB modulate *E. histolytica’s* response to OS and its survival. Almost 50% of coding genes were modulated by the joint action of OS and LB, resulting in cell survival. Firstly, we identified genes for which the OS-induced changes were minimized by pre-incubation with LB (e.g. genes involved in protein synthesis, intracellular trafficking, nutrition, homeostasis, and peroxidases). We found that bacteria exert a “protective” effect on *E. histolytica*, and help the trophozoites to survive OS. Secondly, we identified genes for which changes in expression (upregulation or downregulation) were reversed by pre-incubation with LB (e.g deubiquitinating or glycolytic enzymes). Our results indicate that proteasome activity and glycolysis changes are involved in the stress response of *E. histolytica* in the presence of enterobacteria (e.g. cluster 3 and 4, Fig. 5). Interestingly, previous studies have demonstrated that amoebic glycolysis-related enzymes are inactivated under stress conditions^25–27^ - emphasizing that a reduction in the parasite’s glycolytic activity (its sole source of ATP) is central to the stress response. At this point, the key question is how enteric bacteria are able to control the expression of genes in *E. histolytica* exposed to OS. In fact, the notion of commensalism or mutualism cannot explain what is happening under our experimental conditions, since the bacteria will be phagocytosed by the amoeba and hence destroyed. We have therefore highlighted a complex interrelationship between intestinal microorganisms, whose cohabitation may favour the development of an infectious disease (i.e. amoebiasis). The bacteria not only provide the amoebae with nutrients but also appear to have an unexpected role in helping the parasite to resist OS and establish itself in the intestinal mucosa. One can hypothesize that some bacterial components compensate for the lack of certain amoebic products required to resist OS (e.g. catalase), although this will require further investigation. Nevertheless, the gene response here observed with enteric bacteria does not occur in the presence of *L. acidophilus*. Probiotics (and more specifically *L. casei*) have an anti-proliferative effect on Entamoeba^28^, which seems to be unrelated to the pH changes induced by *L. casei* in the medium. Furthermore, it has been suggested that the probiotic effect of certain bacteria (such as *L. acidophilus*) is mediated by the ability to produce H_2_O_2_^29^ and to maintain a normal, homeostatic microbiota^30^. The combination of added H_2_O_2_ (used to induce OS in the parasite) and H_2_O_2_ derived from *Lactobacillus* may explain the difference in gene expression between trophozoites exposed to *Enterobacteriaceae* and OS and trophozoites exposed to *L. acidophilus* and OS.

### LRR proteins in *E. histolytica*: a first line of defence against bacteria and OS

In addition to our findings on the stress response, we also observed the induction of an antibacterial response in which the amoeba’s LRR proteins appear to be activated by the presence of enteric bacteria. Indeed, this response was absent when *E. histolytica* was incubated in the presence of LB only - indicating that it is not related to phagocytosis. The majority of LRR proteins in *E. histolytica* belong to the BspA family (a total of 98 genes over 137, which were probably acquired by lateral gene transfer); this first class of LRR displays homology with bacterial LRR factors^31^ and those in *Trichomonas vaginalis* BspA^32^. *Entamoeba histolytica*’s BspA comprises 1 to 10 copies of an LRR_5 domain (IPR026906), and two BspA proteins have previously been identified^33,34^. A phosphatase domain is present in a second group of 15 amoebic LRR proteins, and other LRR motifs are found in a third group of 26 proteins). The 137 amoebic LRR proteins identified in the present study all belong to one of these three classes. The most strongly expressed LRR proteins specifically display structural homology with the TLR ectodomain needed for bacterial product recognition. TLRs are microbial sensors that elicit effective cell defence mechanisms in respond to bacterial infection or cell damage^19^. Throughout evolution, these receptors have been conserved in plants, flies and mammals^17^. The absence of transmembrane domains in amoebic LRR proteins indicates that the latter are cytoplasmic proteins or that they need a co-receptor at the cell surface (as observed for TLR-4 in B lymphocytes)^20^. In addition to the amoebic proteins’ structural homologies with TLRs, we also demonstrated similarities in the functional behaviour; as is the case for transcription of the TLR-4 gene when LPS activates leukocytes, the transcription of amoebic LRR proteins is specifically regulated in the presence of bacteria and OS^35^. In humans, TLRs are subdivided into two groups as a function of their cellular localization and ligand specificity. For instance, cell-surfaced expressed TLRs, (e.g. TLR1, TLR2, TLR4, TLR5, TLR6 and TLR10) detect products (such as glycolipids, lipopeptides and flagellin) that are present in a wide variety of microorganisms. In contrast, endosomal TLRs (e.g. TLR3, TLR7, TLR8 and TLR9) are involved in the sensing of nucleic acids. Following ligand recognition, TLRs transduce the signals required for the activation of innate immunity effector mechanisms. Here, we describe amoebic protein homologies with the two subcategories of human TLRs. However, the signalling pathway activated in *E. histolytica* remains to be characterized. Nevertheless, our study is the first to attribute a function to the huge LRR gene family, which includes BspA encoding genes that are well represented in *E. histolytica* and in bacteria. Indeed, bacterial nucleic acids are the best candidates for activating a potentially immune-like response in this ancient unicellular organism.

## Conclusion

Characterization of the human microbiota is providing new insights into the complexity of host–parasite–bacterium relationships. During amoebiasis, the parasite encounters several types of stress as a result of the host’s response to infection. Given that *E. histolytica* phagocytises bacteria in the intestinal lumen, we demonstrated that enteric bacteria can influence the course of an amoebic infection. Intestinal bacteria with the exception of probiotics, in a newly here-discovered cooperative function, efficiently supply multiples factors necessary for amoeba survival boosting the infectious process. The present study provides the first crucial insights into how bacteria may influence the success of infection by *E. histolytica* other than through a contribution to the parasite’s nutritional needs.

## Methods

### Chemicals and reagents

*S*-nitrosoglutathione and H_2_O_2_ were purchased from Sigma-Aldrich, St. Louis, MO, USA.

### Microorganisms

Trophozoites from *Entamoeba histolytica* strain HM-1:IMSS were grown under axenic conditions in Diamond’s TYI S-33 medium at 37 °C^36^. All experiments were performed on trophozoites in the exponential growth phase. *Escherichia coli* O55 (isolate TB182A) was kindly provided by The Thomas S. Whittam STEC Center (East Lansing, MI, USA)*. Lactobacillus acidophilus* strain ATCC 4356 was kindly provided by Dr E. Segal (Department of Biotechnology and Food Engineering, Technion, Haifa, Israel). *Enterococcus faecalis* strain ATCC 29212 and *Salmonella enterica* (clinical isolate) were provided by Dr Y. Geffen (Clinical Microbiology Laboratory at Rambam Medical Center, Haifa, Israel)^37^.

### Exposure of *E. histolytica* to OS or NS

Trophozoites (~1 × 10^6^/ml) were pre-incubated with bacteria (~1 × 10^9^/ml), in serum-free Diamond’s TYI S-33 medium, following previously published conditions^8^, for 30 minutes at 37 ^◦^C. Trophozoites (1 × 10^6^/ml) exposed to OS alone were incubated for 60 minutes in serum-free Diamond’s TYI S-33 medium at 37 ^◦^C supplemented with 2.5 mM H_2_O_2_ (Merck, Germany), which corresponds to the lethal dose, 50% (LD50). For the determination of *E. histolytica*’s LD50 for H_2_O_2_, concentrations of 0, 1.5, 2.5, 3.5 and 5 mM H_2_O_2_ were used. Trophozoites (1 × 10^6^/ml) exposed to acute NS were incubated in serum-free Diamond’s TYI S-33 medium with 350 μM GSNO (Sigma-Aldrich, Israel) for 60 minutes at 37 °C.

For experiments with diverse bacteria the trophozoites (1 × 10^6^) were incubated with: (i) 1 × 10^9^ live *E. coli* O55 for 30 minutes and then with H_2_O_2_ (2.5 mM) for 1 hour (EC); (ii) 1 × 10^9^ live *S. enterica* for 30 minutes and then with H_2_O_2_ (2.5 mM) for 1 hour (S); (iii) 1 × 10^9^ live *E. faecalis* for 30 minutes and then with H_2_O_2_ (2.5 mM) for 1 hour (EF) and (iv) 1 × 10^9^ live *L. acidophilus* for 30 minutes and then with H_2_O_2_ (2.5 mM) for 1 hour (L). The cells were then harvested by centrifugation at 2500 rpm and lysed in TRI Reagent (Sigma Aldrich) according to the manufacture instructions.

### RNA extraction

Total RNA was extracted from control trophozoites, trophozoites pre-incubated with LB or KB and then exposed (or not) to H_2_O_2_ (in three biological replicates) using the TRI reagent kit, according to the manufacturer’s instructions (Sigma-Aldrich USA). Libraries were built using a Truseq mRNA-Seq Library Preparation Kit (Illumina, USA), according to the manufacturer’s recommendations. Quality control was performed on an Agilent Bioanalyzer. Sequencing was performed on a HiSeq. 2500 system (Illumina, USA) and produced 65-base single-end reads.

### RNA-Seq data analysis

Adapter sequences and low-quality sequences were removed from RNA-Seq by applying an in-house program (https://github.com/baj12/clean_ngs). We found that almost 95% of the annotated coding regions had at least one read in all tested conditions - indicating that our dataset was deep enough to analyse the majority of transcripts annotated in public-access databases. Only sequences of at least 25 nucleotides were considered for further analysis. Tophat version 1.4.1^38^ with default parameters was used for alignment against the reference genome (AmoebaDB-1.2, retrieved from http://amoebadb.org/amoeba/). Genes were counted using HTSeq-count version 0.6.1^39^ (parameters: -t CDS -i ID -m intersection-nonempty -s no).

Count data were then analysed using R version 3.2.2 and the Bioconductor package DESeq2 version 1.10.1^40^. Normalization and dispersion estimation were performed with DESeq2 (using the default parameters), although statistical tests for differential expression were performed without applying the independent filtering algorithm. A generalized linear model including (i) the effect of stress (+OS vs −OS), (ii) the effect of bacteria (LB, KB vs. NI) and (iii) the interaction term was set up in order to test for inter-condition differences in expression and to test the interaction between stress and bacteria. For each pairwise comparison, raw p-values were adjusted for multiple testing using the Benjamini and Hochberg procedure^41^. Genes with an adjusted p-value below 0.05 were considered to be differentially expressed.

Principal component analysis and hierarchical clustering were performed using variance-stabilizing transformed counts. Hierarchical clustering of the genes was based on the correlation distance and the Ward aggregation criterion. The average profile was established by computing the mean log_2_-normalized count for the genes in each of the four identified clusters.

The functional classification, GO term analysis and protein class analysis were performed with PANTHER tools (http://pantherdb.org)^42^. We used SWISS-MODEL (https://swissmodel.expasy.org/) for protein structure modelling^43^. The template library was tested for evolutionarily related protein structures that matched the amoebic protein sequence. The top 50 templates were retrieved and those with the greatest sequence coverage and the highest percentage of amino acid identity were used to visualize the 3D structure of amoebic candidate proteins.

### Availability of data

RNA-Seq data have been deposited at the Gene Expression Omnibus (http://www.ncbi.nlm.nih.gov/geo) under the accession number GSE104434.

## Electronic supplementary material

Supplementary Information

Table S1

Table S2

Table S3

Table S4

Table S5

Table S6

Table S7

Table S8
