# Supplementary material for: Enteric bacteria boost defences against oxidative stress in Entamoeba histolytica
Source: Sci Rep. 2018 Jun 13;8:9042. doi: 10.1038/s41598-018-27086-w (PMC5998147; doi:10.1038/s41598-018-27086-w)
Supplement: Supplementary file 1 — Supplementary Information [file 41598_2018_27086_MOESM1_ESM.pdf]

SUPPLEMENTARY INFORMATION (9 pages including this)

From

Enteric bacteria boost defences against oxidative stress in *Entamoeba histolytica*

Hugo Varet, Yana Shaulov, Odile Sismeiro, Meirav Trebicz-Geffen, Rachel Legendre, Jean-Yves Coppée, Serge Ankri and Nancy Guillen

Scientific Reports, 2018

Included material

Figure S1: Effect of Escherichia coli O55 on the resistance of *E. histolytica* to nitrosative stress.

Figure S2: Effect of diverse bacteria on the resistance of *E. histolytica* to oxidative stress.

Figure S3: GO Terms enrichment in *E. histolytica* under stress upon incubation with diverse bacteria.

Document DOC1: LRR motifs in the amoebic proteins identified in this work (see Table S8).

Figure S1.
Effect of *Escherichia coli* O55 on the resistance of *E. histolytica* to NS.

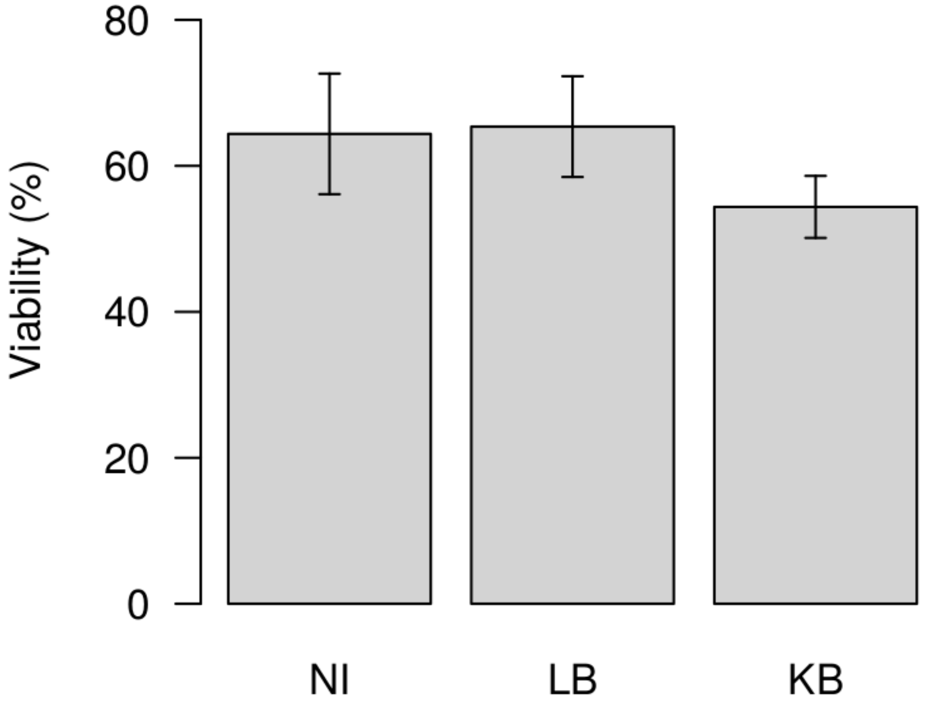

Trophozoites (1×10^6) were treated with (i) GSNO (350 µM) for 1 hour at 37°C (NI); (ii) 1×10^9 live *E. coli* O55 (LB) for 30 minutes and then with GSNO (350 µM) for 1 hour; and (iii) heat-killed *E. coli* O55 (KB, the equivalent of 1×10^9 bacteria) for 30 minutes and then with GSNO (350 µM) for 1 hour. The trophozoites' viability was determined in an eosin dye exclusion assay.

Figure S2. Effect of diverse bacteria on the resistance of *E. histolytica* to oxidative stress

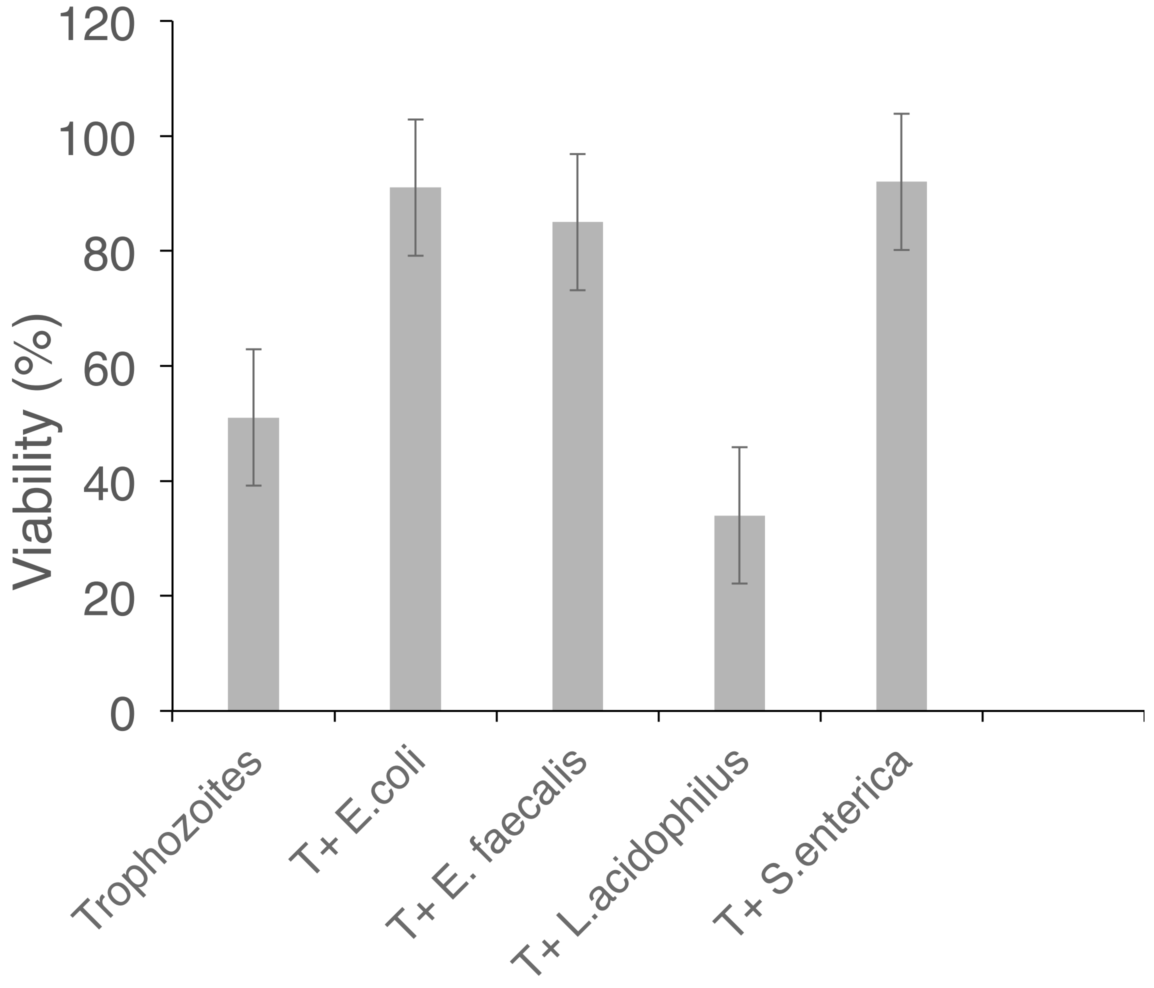

Trophozoites ($1×10^6$) in the presence of the diverse live bacteria ($1×10^9$) were treated with 2.5 mM H_2O_2 for 1 hour at 37°C. The trophozoites' viability was determined in an eosin dye exclusion assay.

Figure S3. GO Terms enrichment in *E. histolytica* under stress upon incubation with diverse bacteria.

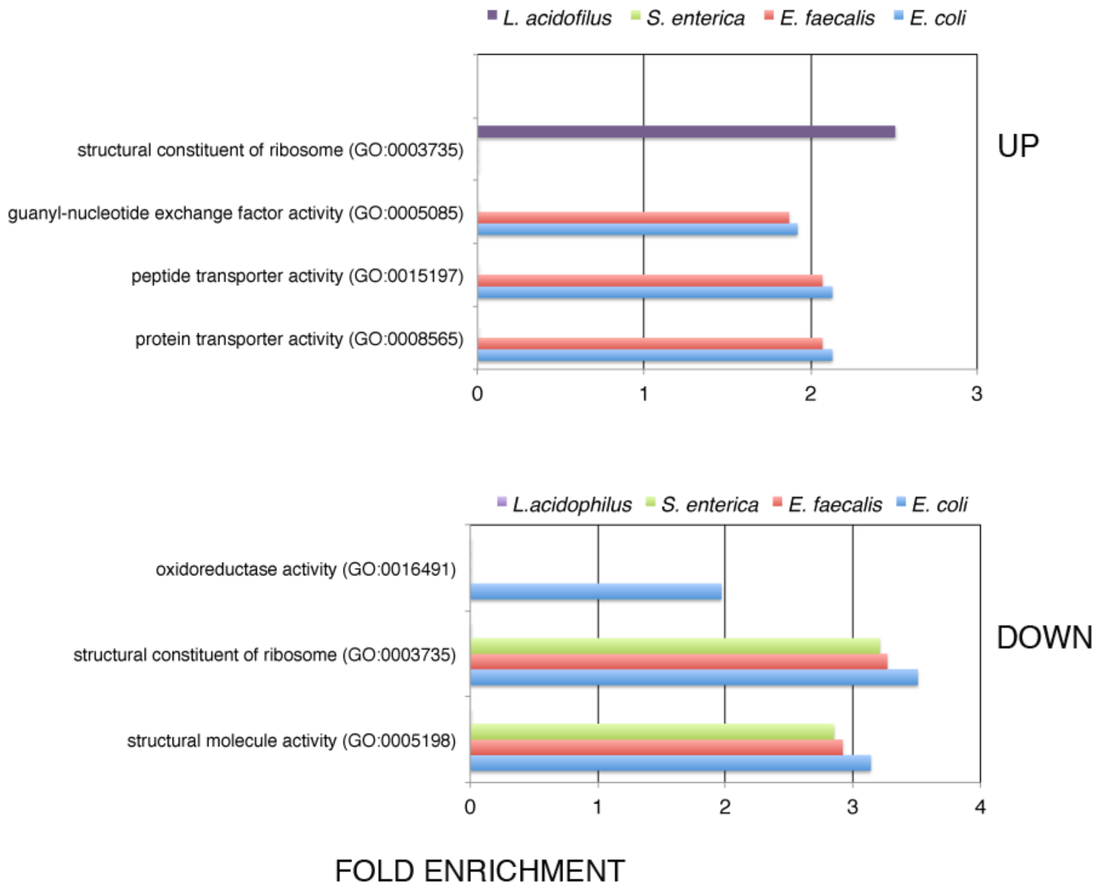

The data from Supplementary Table 6 were analysed with PANTHER tools (http://pantherdb.org/), in order to determine the fold enrichment (FE) of gene sets with GO terms, relative to the background list for all genes in the amoeba genome. The GO terms were used to identify enriched molecular processes; those with an FE ≥ 2.5 were plotted.

Supplementary DOC 1. LRR motifs in the amoebic proteins identified in this work (see Table S8).

The diverse LRR motifs were localized by visual inspection of amino acid sequences. We took into account the consensus sequences for LRR

Bsp A: LxxIxLxxxVxxIgxxAFxxCxx
LRR11: LxxLxLxxNxL
LRR12: LxxLxLxxCxxL

EHI_120570, 356 aa

MQLGYNEIMIVSKYFDDINDFINLEIGVKRFQGNMERFHFNPIPLNQYSRKLFPNIETF
HIYNIYDEVFEDGRIFKYYVIWYKVDYSRYLEEKEEMNEYKNIEYTQEDRKKYGNTIPNE
VTSLGYWCFSLCDDIQEIEIPTSVTEIRSYCFERCSS
LQTITIPTNVSKIGDSCFYKCSS
LQTITIPTNVSKIRECCFYGCSS
LTTINIPPSVTKIGNRCFEECYS
LTSINIED-VKYISEKRIFMNEPV
LISIKILENLTIINGKNIEKKDINEFIIPSSITKLGDYCFEYCYS
LQTINIPTSVSKIGDYCFKECTS
LTSINIPSSITSFGKGCFYHCRCEEELKKNKTIPEYCFEE

EHI_139980, 666 aa

MQRDNPNNMKRIRRKMMSLNIPTRPFLLTENTGSIIPTLSEIILDSFGRFEQNFSSQQE
QTPIALPPVLASKLIYRISNNPNASESDLNIILFRILSSGTPLQVIDLHHTHLLTKFTC
MNLSQLCNNVSITTLNISYATGISSESLKVLFRSFKQLKYLNTEACVGFDDNAFISLLT
IHPPLEVIVISNCPKISDKSVIELKNINSLISFKANNIQLTISSISVLHGLKEIELMGN
NCLDDQCLLKISQLNPDLTRICFGNSRLSDEALQRLLQQLNGNLISLNLSGCTRAGPMT
LAQLFNSQFKLKYLNLANCFGINGDSILESYHPMFRSTLFRWLEEIEYLNISGCIRLSE
EFITTTLIASPQLRSLILDDTAVTDQCLQEFIQSSITYNQTVRRSIHTPGMKVHPLTLS
LKRCIRLTDNAYTNLFQTRGVDLISLNLSFCNSLSSLSLNVFSLYSNTLTSFYASNNDM
ISEYSWLKFIASCPQLRVLFLSNNRGITNEVINQIKISCPLISHLDISSCLSINQFVIP
ILCKMQSLEYIDISFDVMINEDGIISLVNSLTRLSTFCMQGIAFKQSYLFTEEAQWLNS
LKLNFIPMCGDDVMRNIASYCPLLTSVELRMCSGVTDNGIEMLLQKCTKISHLVLGGTS
ISQFKMMELVGRGLFIA

EHI_087890, 484 aa

MSSDKQHYNKKNMGIKQFPKDCISQASIITSIDLSNNEIINLPKEMGPFKVLTHFRMMA
NKVSSLPLSFTTLTNLRHLDLNANCFTEFPTQITSLTNLEEIQMIQNQLTSIPDCIGNL
VKLQRISFTANFLKSLPKGLAKCVDMNYIELTSNEFEEFPDVICELRKVTILMLQQNRI
KEVPDSISKLEKLSGLYLSSNNFGKFPESVCTIPSLTQLELDNNNFVDIPDSLSQLTKL
KTLIINKSFISCLNSVDMMSNLCQIVLSDTKCMFLPDLSQNSKLTSLNVIRGYLNEVKS
LPPNCSCRFSNNQIESIELPENGVLQYMILSNNRLKVSPNLSMLSKISRLDISQNRITR
FNENTCHPTLQQLDISCNPLVEFPVCITKCQSLKILNLSDCHLYDIPSNVLSSLSNLET
LYIGCNHLSSLESLSVLKKLRALYLQSNNLLHFPQSIFDLITLKTLFVSNNYITTIPNE
ISQLTQLEQLAP

EHI_154170, 253 aa

MKIGYNEIMIISKYFDDINDFINLEMRVKRFQGNMERFHFNPIP
LNQYSRKLFPNIETFLIYNEKDEIFEDGRINNYVIWHKVKYRVIYKGTIPPEVTSLGVE
CFSNYKDLITFRIPTIINKLGVSCFHGCNEERIFVNETILISIEIPRNPEIINRKNIEK
KNINAPIIPTTISKLGDYRFEDCTT
LRLIIIPTSVSELGEYCFINCTS
LTSIDIPTTVSEIGNGCFDGCFRKKNEKEQNVKENI
LSKILYK

EHI_137910, 1065 aa

MFIILYFFIYNCFGKCELNFTSASRYGLNGLIVISSNTRECSGDIKIPNSGIDPISNEE
KRVEVIGWNAFSNS
LIKSIEIPTSIKIICLNAFSNCNN
LQNINIPKSVYRIDYQAFSNCKS
LVSINFNNAEIKELGNELFIGCNK
LKSIDLSTFSIETIYEDTFNGCSN
LINVILPYTLREIKESAFSGCTS
LTTINLHNTLINSIKEKAFKKCTS
LITITFPSTLTSLSNNSFEKCTSLTSIIFQSVLSSSNNNIPFNECNKLKSIYYTGTDLF
TNCIFSNNPIQNIYVNSEYQQDFICSIKVIKNINHSPIIYLCKYSDSNECVSYLSYRKS
SINQLTNNEICYQRGSTNQKIIINDNKYYLVDCNNNTKIQYYQNKEINNNIISNFIFSV
GYKEINDNNIHILREYFISKRIDNIQWITNQCTVTKHLYLNENENSSIKTKDIIIEIYR
NGYILNNGIFYIEKNIKMNEIQTLYFGNEQMYREVLINDYFESMNQENEYIFECISKDL
CKTYKFNTTNNLFIEDIDFPLLKKEIKTNILAIGYEYFHFYYSPYFSQQVKFTNYCENG
KIFRTIGMELQLMSNFVNENECPKDDYSSFSIEIESKNETIEINSRIYKISVLDNRISD
STPIYLKFTNSYLRVIPNKCFQITNSSSLLFIPGIYGDKNYIFLKEYNSTNNCNGIYGI
SEFSPDQYSYTKFNINDDYSIISYFDNDYKDCGINKIEEASLIRYVLPDINKCQFKSTY
TINEFNLIETKYDNSDCKGNIIIQESYALKRCINDTNQLHIIIATFPIKSEDCNNKFKG
CINCDLDECYECPSGYGLSMKKDKCIDCSIFTNDCIQCNNQTCTKCNSIDNEIRVYHNN
NCLTCKEAFNDNGCLECNVNNCTLCSSINGTLRYYNNGSCKTCEEAYDENCSDCDGIEC
KKCKIEYGLKIGRHGCMKCFDLDSKCLECGEGICTKCYNNLLFKNNKCQSCDDIYGKDC
LSCDINNCLTCDNNKVVIESKCISCNKLFDHCVHCNTTHCLDCDSIAYSLSNGKCINQN
NEQGNNSCLISILLVFSIILFFLI

EHI_017710, 426 aa

MSDHQIEKKP
LTNLTLRQYVFKATEPIAVEVPEELTNLEEMEFFSCVN
LDKLILSPHMLTIPSGLCWGCVMLTEVILPSCLRSIEDNAFKSCIR
LKTISLPPTITHLGSNSFNDCES
LQKITLSSIKKISTR-AFCGCFS
LKEVELPECLESIDESAFALCRS
LDKISIPNNVSSIGKMAFNECSS
LTAISLPQNLQLFDVSALMRCNKLQRIILHGKNEINFKVSYHVSQLLEENGITCNNIEL
INGDDLIFSCNEIPSSISSIGDSYYKECEFKTTSFIIPSTITSLGESAFCLCGN

LKK**I**V**I**SSSITNLPDYC**F**AY**C**TD
LIE**I**K**I**PTSITS**I**GTRCFHN**C**IS
LKE**I**YVPDS**V**IH**I**GESC**F**SN**C**TK
LTS**I**KIPTTCIEKENLSQVFLGSKKLSSINGNSFDYTDECLCDEFIEEDDNDEIHAQGW

EHI_123820 500 aa

MQLGYNEIMIVSKYFEDINDFINLEIGIKRFRGNMERFHFNPIPLNEYSRKLFPNIETF
HIYNGYDKIFEDGKIFKYVIWYDVSYSRYLEEKKEKNEYKNIEYTIYDREEYGNTIPIE
VNSLGINCFYRCNDIQSINIPTSVSKIGNECFYQCTS
LTSINIPTSISEIGDKCFCGCYS
LRTIDIPISISKIGYCCFSGCSS
LQTINIPTNVSKIEYWCFKYCTS
LKSINIPTSISEIGNGCFEECSS
LTSINIEDVKYISEERIFMNEPV
LISIEIPKNLKMINGKHIEKKDINEFIIPSSITEIGDYCFKECLS
LKSIDIPISISKIGYWCFCECSS
LTSINIPTNVSKIGDYCFYGCYS
LRTIDIPTNVSKIGYCCFSRCSS
LKSIDIPTSVTEIGYWCFCECSS
LRTINIPTSVSEIGNYCFYGCSS
LTSINIPTNVSKIGGGCFYKCTS
LISINIPSSITSFRNGCFYGCGCEEELKKNKSIPEYCFEEYFYEEIR

EHI_128460, 237 aa

MQLGCNEIKIVSKYFKDINDFINLEMGVKRFRGNMERFHFNPIPLNQYSRKLFPNIETF
HIYNKEDEIFKDGKIFKYVIWYKVNYSKYLKEKEEGNICKNIEYTKEDREEYGNTIPIE
VNSLGNRCFRWCGDIQSINIPTNVNELGNGCFYECSS
LTSINLPPSVSKIGDECFYRCSS
LTSIKIPTSVGKIGEYCFYDCSS
LKSIEIPPRVNKIREGCFCNCTSLTSVTMTSSVTSF

EHI_176480, 241 aa

MQLGYNEIMIVSKYFEDINDFINLEMGVKRFEGNMERFHFNPIPLNQYSIEFFPNIETF
HIYNEEDEVFEGGRIFKYVIWYKVSYSRYLEEKEKGNVCKNIEYTEEDRNTYGNTIPIE
VNSLGDLCFYGCKS
LQSINIPTSVSKIGNRCFKYCST
LTSINIPTSISKIEDYCFEYCKS
LTSIKIPTSVGKIGNKCFSFCTS
LTSINIQSSITSFGKGCFYRCGCEEELKKNKQIPKYCFEK

EHI_134140, 439 aa

MALGYNEIMIVSKYFEDINDFINLEMGVKRFRGNMERFHFNPIPLNQYSIEFFPNIETF
HIYNEYDEIFEDGRIFKYVIWYDVSYSRYLKEKEEMNEYKNIAYTEEDRIKYGNTIPIE
VNSLGINCFGQCDIQSINIPTNVSEIGDECFYRCTS
LTTINISTSVSEIGNKCFYGCLS
LTTINIPTSVIEIGNKCFYECKIQSITIPTSVNEIGNECFKYCKS
LTSIEIPTSVSKIGDGCFEYCTS
LTTINIPTSVNEIGNECFKYCKS
LTSIEIPTSVIEIGNECFKYCKS
LTSIEIPTSVSKIGNECFSFCSS
LTSIEIPTSVSKIGNKVFYGCDDIQSINIPTSVIEIGNECFKYCKSLTSIEIPTSVSKI
GDGCFEYCTSLTSITIPTSVNEIGNE
CFKYCKSLTSIEIPTSVIEIGNECFKYCKSLTSIEIPTS

Only with *Salmonella enterica*

EHI_119470, 574 aa
MKHTLNIDSIQQVALYFETIDDYKNIILSSKKFQHLLEYFKSNPIPLNDETIKFFPNIN
TYNLFSLQDTFYESLSSLKICFYCRISYQRSLKEIKKGNTCKFIYYSKEDKKKFGDILP
SQVKKIGKECYCNSPS
LSIFEVPYSIVEIGDKSFMNCTS
LTSIVLSSSITRLCNYTFHKCIS
LKKIKLPQSLKHIGYNCFSGCSS
LNSIQLPEMLLSIGDYCFDFCVN
LTSLAIPSSITYFGIQCFCNCSSLKSLSIPSNLTSFNRQQFYGCSSLEEINVPAPYWIV
GKHLFFNKMKLVGCLPSTINSINKIPVAFDMNQTSFEVPTEVKSFGNKCFQDEHS
LRSISIKKFIDRLDNYCFEGCLLVTSISLPSTIKVIENGCFCGCIS
LKEINIPSSLTRLGDYCFYRCSSLSAVSLPSSLKVIPYQCFSGCSS
LTSIDLPQGVLKLSDQSFKDCAT
LQSINIPSTITKIEVGCFNNCLRLSEVSFYSPIQKIPDFCFSNC
LQTITLPLSVISIGNRSFCNCVK
LQSIQIPSLTTSLGNDCFTNCSSLVSLGSLPKNAILGDDCFFGCEKLFY

EHI_127100, 482 aa
MSSEESITLSSCSSTSTAYSEQIQLAIAETDTFGFFSFEVDPLFLVAQYLQTESDFINF
IQISKRFQVVLSYFQSNPISV
NSLRLFPQIKTQYLFNKNDKRIKGVSKYVVYRCLLTHTEAKKLNRSNVSFKRVVFKKDKN
AKKHHIPKLTTDLADCCFTKSVY
LKSILIPSTVTLLGSDCFLDCSS
LSKLVLCSSLIHLGISSFENCKS
LRHVVLPLLIKEIPDNCFKECTQ
LKDVVLPSCITRIGHSSFENCLS
LTEIINTSNIIELGQEAFKGCHS

LKQLHFSSSLNYIGVSCFDDCDL
VKINPPPLSSLKCITNKQSLDNKTPSSNHIVLTSDDCIEIMEEIPLTINVIGRSAFKYI
MPSQLILPNSITRIESNAFYQCTL
LTSIIFSTSLKVIESRAFMGCSH
LKSISLPASIQFIGDEAFNNCYE
LTSIVVEGFPCIG-YHAFSHCNELQNIIIPPFSNQNTNDIFFGIKIPSK
PCCIV

EHI_112290, 213 aa
MENTTKRNDKERMRLGYNEIMIVSKYFEYINDFINLEEREKKETNECKNIEYTQEDRNT
YGTTIPIEVNSLGDICFRWCDDIQSINIPTNVSKIGDKYFEYCTSLRIINIPTNVSKIG
DKYFERCSSLTSNSYKNIQFVGEDRIIVNEQIMASIPIGRLKIINEKEIKPKNINEFVI
PTTVTKLGNYCFENCYS
LTSIKIPTSVSEIGKECFL

EHI_041470, 448 aa
MQLGYNEIMIVSKYFEDVNDFINLEMGIKRFQGNMERFHFNPIPLNQYSKLFPNIETFH
IYNKYDEIFKDGKIIKYVIWYKVEYSRYLKEKEEMNECKNIEYTEEDRKKYGNTIPIEV
NSLGINCFYGCNDIQSINIPINVSKIGECCFLCSS
LKSINIPTSVSKIGDSCFSGCLS
LTSITVPPSVTKIGYGCFDGCSSTSINIPTSVSEIGYGCFDGCSSLTSINIEDIKYISE
ERIFMNEPVLISIEIPKNLKMINGKNIEKKDINKFIIPSTITKLGYECFEGCSSLI
LINI--PTSVE–IGNECFYGCES
LKSIEIQTNVTKIGNECFEYCKS
LTSINIPTSVIEIGNC-FRECTS
LTSIEIPTSVNEIRNTCFGWCESLILINIPTSISEIGEYCFYECSS
LTSINIPTSFTSFEICCFYHCGCEEKLKKNKKIPKYCFKKY
